# Family Cohesion and Preschool Children’s Prosocial Behaviour in China: The Mediating Effects of Parent–Child Relationships and Parenting Sense of Competence

**DOI:** 10.3390/bs16030347

**Published:** 2026-02-28

**Authors:** Xu Chen, Jing Chen, Xin Gao

**Affiliations:** 1 Normal College, Jimei University, Xiamen 361021, China; chenxu@jmu.edu.cn (X.C.); chenjing7@jmu.edu.cn (J.C.); 2 College of Education, Zhejiang Normal University, Jinhua 321004, China

**Keywords:** family cohesion, prosocial behaviour, parent–child relationship, parenting sense of competence, preschool children

## Abstract

(1) Background: Prosocial behaviour serves as a crucial indicator of mental health development among preschool children. This study aimed to reveal the path through which family cohesion affects prosocial behaviour in Chinese preschoolers, while exploring the potential roles of parent–child relationships and parenting sense of competence in this relationship; (2) Methods: The questionnaire method was used, with 515 parents of preschool children from Southwest China participating in this survey (115 fathers, 400 mothers; Mage = 34.37, SD = 5.31). (3) Results: The key findings of the study are as follows: 1. Family cohesion exerted a direct and positive significant effect on the prosocial behaviour of preschoolers such that strong family cohesion effectively promoted children’s prosocial behaviour; 2. Parent–child relationships and a sense of competence in terms of parenting played a joint mediating role in the relationship between family cohesion and preschooler’s prosocial behaviour. Specifically, strong family cohesion enhanced the quality of parent–child relationships, which in turn improved parents’ parenting sense of competence, ultimately facilitating the development of prosocial behaviour in preschool children; 3. Further propensity score-matching analysis revealed that the influence of family cohesion on the prosocial behaviour of preschoolers varies by parent, with parenting sense of competence being significant among fathers and parent–child relationships being significant among mothers. (4) Conclusions: In educational practice, greater attention should be paid to the role of fathers in Chinese family education, especially regarding their parenting capabilities and experiences. Additionally, it is essential to encourage Chinese fathers to actively fulfil their parenting duties and responsibilities.

## 1. Introduction

### 1.1. The Development of Prosocial Behaviour in Preschool Children

Children’s prosocial behaviour refers to children’s spontaneous expression of care and empathy for others, as well as behaviours that are beneficial to others in social interactions (Eisenberg et al., 2015). These behaviours are an external manifestation of the development of personality and moral quality, and they are an important aspect of children’s social interactions. Additionally, they are an important indicator of children’s social development. Prosocial behaviour occurs in infants, and individuals undergo rapid development during the preschool period (Hashim et al., 2023; Vanderbilt et al., 2023). Empirical research has shown that in early childhood, children who often exhibit prosocial behaviours are more likely to be accepted by the group and less likely to be prone to problem behaviours such as aggression (Hay et al., 2021; A. Li et al., 2023). In addition, the development of children’s prosocial behaviour is closely related to their academic performance (Armstrong-Carter et al., 2021; Puccioni et al., 2020), interpersonal relationships (Jambon & Malti, 2022; Grueneisen & Warneken, 2022), and psychosocial adaptation (Stacy, 2020).

In summary, on the one hand, preschool is a critical period for children’s prosocial development, and investigating the factors that influence preschool children’s prosocial behaviour plays a crucial role in fostering positive social relationships. On the other hand, the development of prosocial behaviour depends on families. As the initial and long-term spaces for the social development of individuals, families are a pivotal contextual factor in children’s growth. Therefore, the present study investigates the mechanism through which family cohesion affects the prosocial behaviour of Chinese preschool children.

### 1.2. Relationship Between Family Cohesion and the Prosocial Behaviour of Preschool Children

In accordance with ecological systems theory, the family stands as one of the microsystems exerting the most substantial influence on individual development (Bronfenbrenner & Ceci, 1994). As a significant indicator of the family atmosphere, family cohesion refers to the degree of emotional connection between an individual and other family members (Olson et al., 2010), which is an inherent characteristic of the family system. A number of studies have shown that family cohesion is closely related to children’s prosocial behaviour, i.e., as family cohesion deepens, children’s prosocial behaviour increases (Miller-Graff et al., 2020; Hur et al., 2017; Manyeruke et al., 2021). In addition, one study showed that father–child cohesion, but not mother–child cohesion, can positively predict children’s prosocial behaviour and that prosocial behaviour affects mother–child cohesion but not father–child cohesion through loneliness (W. Chen et al., 2022). In conclusion, a significant correlation may exist between family cohesion and the prosocial behaviour of preschool children. Moreover, when considering fathers and mothers separately, family cohesion could have varying impacts on children’s prosocial behaviour.

### 1.3. The Parent–Child Relationship and Parenting Sense of Competence as Mediators

The parent–child relationship is a family relationship established by parents and children based on blood relationships and genetics, and it is also the earliest interpersonal relationship established by an individual (Bronfenbrenner & Ceci, 1994). One study showed that, compared with family structure, family cohesion and the parent–child relationship were more strongly correlated with children’s prosocial behaviour (Herke et al., 2020). As one of the main relationships among family members, the parent–child relationship is an important reflection of family cohesion. Several studies have shown that the parent–child relationship can directly or indirectly predict children’s prosocial behaviour (Zhang et al., 2022; Ferreira et al., 2016). It can be inferred that the parent–child relationship is likely to play a mediating role in the relationship between family cohesion and children’s prosocial behaviour.

Parenting sense of competence may potentially be regarded as another key factor that bridges the gap between family cohesion and children’s prosocial behaviour. Parenting sense of competence is defined as the efficacy of parents in meeting the diverse needs of young children during the parenting process, which reflects parents’ perception of their capacity to take on parenting responsibilities and their level of satisfaction with their successful implementation of parenting behaviours (Ngai et al., 2007; Oltra-Benavent et al., 2020). As one of the most important dimensions of family functioning, parenting sense of competence may be closely related to family cohesion. One previous study revealed that the greater the family cohesion, the greater the satisfaction with and perceived efficacy of an intervention for parents (Jiménez et al., 2019). In addition, the three dimensions of parenting sense of competence, i.e., efficacy, satisfaction, and controllability, all had a significant positive correlation with family cohesion (Nunes et al., 2016). According to the conceptualization of parenting sense of competence, efficacy refers to the parents’ perceived competence and capability in handling their children’s issues and daily parenting tasks; Satisfaction, an affective dimension reflecting parenting frustration, anxiety, and motivation; and controllability refers to the parents’ perception of being in control of their child’s behaviour and the extent to which they feel they can influence their child’s development (Nunes et al., 2016; Johnston & Mash, 1989; Campis et al., 1986). For example, parenting sense of competence efficacy is related to children’s social-emotional regulation. Studies have shown that parents’ parenting efficacy can not only reduce children’s problem behaviours but also increase children’s prosocial behaviour (Trecca et al., 2022; Jegatheeswaran et al., 2023). Therefore, parenting sense of competence is closely related to children’s prosocial behaviour and may play a mediating role in the relationship between family cohesion and children’s prosocial behaviour.

### 1.4. Current Research

Chinese young children are brought up in a collectivist culture that places emphasis on the intimate interactions between the individual and other people, as well as the execution of altruistic behaviours. Therefore, the development of prosocial behaviours in Chinese children may differ from that in children from other cultural groups. However, research on preschool children’s prosocial behaviour in China started late, and the research results are relatively limited. Moreover, the potential mechanisms underlying young Chinese children’s prosocial behaviour are still unclear. To compensate for this deficiency, the current study seeks to elucidate the mechanism through which family cohesion influences the prosocial behaviour of Chinese preschool children, while also exploring the mediating effects of the parent–child relationship and parenting sense of competence within this relationship.

**Figure 1:**
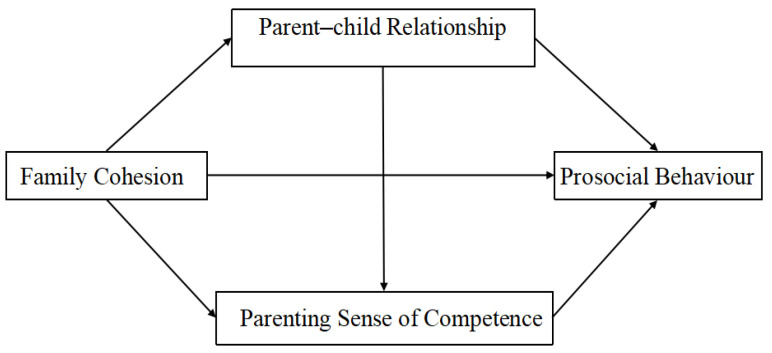
Proposed model in this study.

Based on the literature review presented above, the present study hypothesizes the following: Hypothesis 1: Family cohesion has a definite influence on the prosocial behaviour of preschool children; Hypothesis 2: The parent–child relationship plays a mediating role in the effect of family cohesion on preschool children’s prosocial behaviour; Hypothesis 3: Parenting sense of competence plays a mediating role in the effect of family cohesion on preschool children’s prosocial behaviour; Hypothesis 4: Family cohesion indirectly influences preschoolers’ prosocial behaviour through the chain mediation of parent–child relationship and parenting sense of competence. See Figure 1 for the proposed hypothetical model.

## 2. Materials and Methods

### 2.1. Research Participants

The participants of this study were parents of children enrolled in the same kindergarten. A total of 515 parents of kindergarten children from Southwest China participated in questionnaire survey. Among them, there were 115 fathers and 400 mothers with a mean age of 34.37 years (SD = 5.31). Among the preschool children, 274 were boys, 240 were girls, and 1 was not able to provide their gender. The mean age of the young children was 5.20 years (SD = 1.09). All parents completed the questionnaire after being informed of the study’s purpose and consenting to participate.

### 2.2. Measurement Tools

Family Cohesion: The cohesion subscale of the Chinese version of the Family Cohesion and Adaptability Scale (FACES I-CV) was used to measure the family cohesion of Chinese preschool children. The Chinese version of this scale was revised by Fei et al. based on the Family Cohesion and Adaptability Second Edition (FACESII) developed by Olson et al. (2010). The cohesion subscale consists of 16 items (e.g., “In times of difficulty, family members will try their best to support each other”, “All family members gather together for activities”), all using 1–5 (not = 1; occasionally = 2; sometimes = 3; often = 4; always = 5) for scoring. This scale has been validated as suitable for measuring the cohesion of Chinese families (L. J. Li et al., 2024). In this study, the internal consistency reliability of this subscale, measured by Cronbach’s α coefficient, was 0.833.

Parent–Child Relationship: A one-item statement format was used to ask parents to rate the quality of their relationships with their children on a scale of 1–5 points, where 1 means “strongly disagree” and 5 means “strongly agree”. This one-question measure of parent–child relationships comes from the Chinese Family Panel Survey and is commonly used in China (Institute of Social Science Survey, Peking University, 2018).

Parenting Sense of Competence: The parenting sense of competence scale was originally developed by Gibaud-Wallston et al. In 1989, Johnston et al. developed a scale with an interpretable two-factor (efficacy and satisfaction) structure. This study adopted the Chinese version of the parenting sense of competence scale revised by Yang et al. (2014) to measure the parenting sense of competence of a group of parents of young children. This scale has been proven to be suitable for the population of Chinese parents of young children (Yang et al., 2014; X. Y. Li et al., 2021). The Chinese version of this scale includes a total of 17 items divided into an efficacy subscale and a satisfaction subscale. The efficacy subscale was used to measure parents’ perceptions of their parenting skills and contained eight items (e.g., “I am convinced that I have the skills to be a good mother/father.”). The satisfaction subscale was used to measure parents’ comfort level and contained nine items (e.g., “Being a mother/father makes me nervous and anxious”), with a total score of 9–54 points. Each item in the parenting sense of competence scale was divided into six levels from “absolutely disagree” to “absolutely agree” (1–6 points). Nine items (2–5, 8, 9, 12, 14, and 16) were reversely scored. The total score was 17–102 points, with a higher score indicating greater overall parenting sense of competence. In this study, the Cronbach’s α coefficient of the internal consistency reliability of the Chinese version of this scale was 0.814.

Prosocial Behaviour: The subscales of the Strengths and Difficulties Questionnaire were used to measure young children’s prosocial behaviour. The Strengths and Difficulties Questionnaire (SDQ) was revised and refined by the British researcher Goodman (2010). This scale is a brief emotional and behavioural screening questionnaire that assesses prosocial behaviour, peer interaction problems, emotional problems, and hyperactivity and conduct problems. The SDQ is suitable for children aged 3–16 years and is widely used in most countries worldwide, including China (C. B. Chen & Chen, 2017). This study mainly used the parent version, with a total of 25 items (e.g., “Frequent lying or deception”, “Being teased or bullied by other children”), in which parents evaluated the prosocial behaviour of their preschool children. In this study, the internal consistency reliability of the prosocial subscale, Cronbach’s α coefficient, was 0.760.

### 2.3. Data Processing

SPSS 25.0 was used for statistical analysis and to process the data collected in this study. The survey included (1) descriptive statistics on demographic information, (2) analysis of the relationships and differences among the core variables, and (3) testing of the chain mediation model proposed in this study.

## 3. Results

### 3.1. Analysis of the Family Characteristics of Chinese Children’s Prosocial Behaviour

To explore whether sociodemographic groups in Chinese culture also influence preschool children’s prosocial behaviours, in addition to the variable of interest, difference tests were performed on parental roles (father, mother), parental educational levels (primary school to postgraduate degrees), and marital status (married, divorced, single, remarried, and widowed). A *t* test was used to test group differences in the core variables according to parental role. The results showed that there were significant differences between the father and mother groups in terms of parent–child relationships and the prosocial behaviours of preschool children, with mothers reporting significantly higher scores than fathers (t = −2.81, *p* < 0.01; t = −2.69, *p* < 0.01); (Mmother > Mfather). Next, we used the F test to test the differences in the core variables across educational levels and marital status.

The results showed that there were significant differences in family cohesion, parenting sense of competence, and young children’s prosocial behaviour according to parental educational level (F = 3.25, *p* < 0.05; F = 5.91, *p* < 0.001; F = 2.84, *p* < 0.05). The results reported by parents with a college degree and above were significantly greater than those reported by parents with less than a college degree. In addition, family cohesion and young children’s prosocial behaviour differed significantly according to the marital status of their parents (F = 7.82, *p* < 0.001; F = 4.12, *p* < 0.01), with married parents reporting greater family cohesion and more frequent prosocial behaviours in their children.

### 3.2. Correlations Between Family Cohesion, Parenting Sense of Competence, the Parent–Child Relationship and Preschool Children’s Prosocial Behaviour

The results, in Table 1, of the Pearson correlation analysis showed that family cohesion, parenting sense of competence, and the parent–child relationship were strongly positively correlated with preschool children’s prosocial behaviour. Notably, the correlation between family cohesion and children’s prosocial behaviour was particularly strong (r = 0.29–0.45, *p* < 0.001). A higher frequency of prosocial behaviours in young children is fundamentally connected to enhanced family cohesion, a stronger parental sense of competence (parenting sense of competence) in child-rearing, and positive parent–child relationships, with family cohesion playing a particularly vital role.

In addition, family cohesion, parenting sense of competence, and parent–child relationships were significantly and positively pairwise correlated (r = 0.23–0.50, *p* < 0.001). In families with strong cohesion among family members, parents also demonstrated higher parenting sense of competence and maintained better relationships with their children.

### 3.3. Model Validation Results

Model 6 in Process 3.3 was used to test the hypotheses proposed in this study. After controlling for parental role, marital status and educational level, the results showed that the direct path in the model (family cohesion → prosocial behaviour of preschool children) was significant. Family cohesion exerts a notable and positive impact on the prosocial behaviour of Chinese preschool children (direct effect value was 0.31, and the confidence interval did not include 0). Stronger cohesion among family members inspired more altruistic behaviours in Chinese children. In addition, the indirect paths were effective: mediating path 1 (family cohesion → parent–child relationship → prosocial behaviour), mediating path 2 (family cohesion → parenting sense of competence → prosocial behaviour) and mediating path 3 (family cohesion → parent–child relationship → parenting sense of competence → prosocial behaviour) were all significant (the effect values were 0.04, 0.07, and 0.01, respectively, and the confidence interval did not include 0). Additionally, mediating path 2 had the strongest effect; see Table 2 for details.

This finding indicates that although family cohesion can also have an indirect influence on prosocial behaviour, this indirect effect is chiefly realized through parenting sense of competence. Specifically, family cohesion does indeed indirectly affect preschool children’s prosocial behaviour through parent–child relationships and parenting sense of competence. Strong family cohesion strengthens the parent–child relationship and enhances parents’ ability to successfully raise their children, which in turn fosters the development of more prosocial behaviours in young children. In this process, parenting sense of competence serves as a crucial bridge.

### 3.4. Results of the Cross-Group Test for the Father and Mother Groups

Although the present study revealed the mediating effect of parenting sense of competence, this variable itself displayed significant differences between fathers and mothers. Therefore, the hypothetical model proposed in this study was further explored based on the father and mother groups to clarify the group differences in the mechanisms of young children’s prosocial behaviour. Propensity score-matching (PSM) was used to match participants based on parental role (1 = father; 0 = mother) and with parental educational level and marital status as control variables. The analysis results indicated that a total of 103 pairs of effective samples were precisely matched. Then, mediation model testing was conducted on the fathers (103 fathers) and mothers (103 mothers).

**Figure 2:**
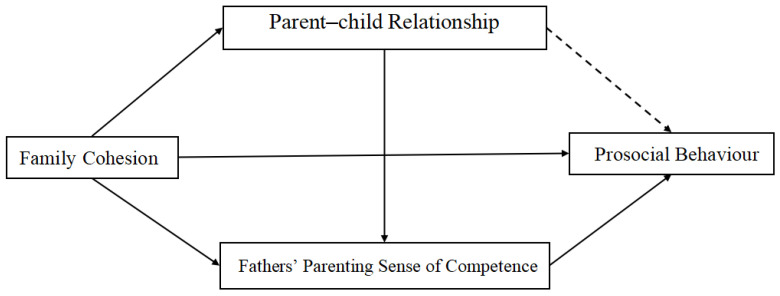
The mediating model in the father group.

**Figure 3:**
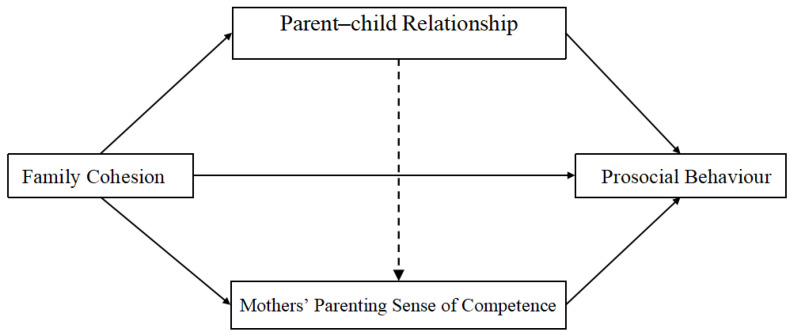
The mediating model in the mother group.

Testing results showed that mediating path 1 existed only in the mother group (for which the mediating effect was 0.07 and the 95% confidence interval did not include 0), while mediating path 2 were present only in the father group (for which the mediating effect was 0.01 and 0.02 and the 95% confidence interval did not contain 0). However, mediating path 3 existed in both the father and mother groups (for which the mediating effects were 0.19 and 0.07, and the 95% confidence interval did not contain 0)—see Figure 2 and Figure 3 for details.

These results indicate that the effect of family cohesion on young children’s prosocial behaviour differs for fathers and mothers. For mothers, the parent–child relationship and parenting sense of competence did play a role in the development of prosocial behaviour among preschool children; however, this influence was relatively minor. For fathers, their evaluation of parenting sense of competence was more critical. If fathers regarded themselves as proficient in parenting, they could stimulate more prosocial behaviour in their young children. Moreover, the chain-mediated effect between parenting and parenting sense of competence was also statistically significant among fathers. That is, the mediating effect of parenting sense of competence in fathers can also be fulfilled through harmonious parent–child relationships. Enhanced family cohesion strengthens the parent–child relationship, which in turn increases parenting sense of competence levels in fathers and ultimately encourages more pro-social behaviours in young children. Comparatively, individual mediators of parenting sense of competence play a stronger role than chain mediators.

## 4. Discussion

### 4.1. The Effect of Family Cohesion on the Prosocial Behaviour of Chinese Preschool Children

This study has verified that family cohesion has an impact on the prosocial behaviour of Chinese preschool children. A greater extent of cohesion among family members elevates the likelihood of preschool children providing assistance to others. The underlying mechanism is that the behavioural development of preschool children stems largely from the imitation of role models. According to Bandura’s social learning theory, children’s behaviour is acquired through indirect observation. For preschool children, adult family members, including parents, are the main role models and objects of observation for social behaviour learning, and preschool children are more susceptible to the influence of family members. Preschool children observe the communication behaviours and interaction patterns between parents or between parents and grandparents, and then they apply these learned behaviours and patterns to peer interactions or groups other than family members (Lansford et al., 2023; Giner Torréns & Krtner, 2019; Stengelin et al., 2023). Additionally, young children hailing from families characterized by a higher level of cohesion are more prone to receiving assistance and experiencing supportive behaviours from family members. This experience of being assisted undoubtedly heightens the young children’s inclination to assist others and spurs more prosocial behaviours (Shah et al., 2021). Therefore, family cohesion is a pivotal factor in the cultivation of preschool children. It is necessary to pay additional attention to the important influence that harmonious family interpersonal relationships exert on the social development of young children.

### 4.2. Mediating Effects of the Parent–Child Relationship and Parenting Sense of Competence

This study revealed that the parent–child relationship and parenting sense of competence play important mediating roles in the relationship between family cohesion and preschool children’s prosocial behaviour. These findings support the study’s hypothesis proposed in the present study, as well as the findings of J. Zhao et al. (2018) and Poulain et al. (2019). The reason for this is that, on the one hand, as an interpersonal relationship among family members, the parent–child relationship can indirectly reflect the degree of family cohesion (Ma et al., 2021). From the perspective of attachment theory, a positive parent–child relationship promotes children’s secure attachment to their parents, resulting in a stronger sense of connection, a greater ability to perceive, pay more attention to and recall others’ well-intentioned information, and a greater inclination to adopt positive attitudes and responses toward themselves and others (Z. Li et al., 2024; F. Zhao et al., 2020; Lee, 2018). On the other hand, parenting sense of competence stems from parents’ sense of efficacy in and satisfaction with their ability to meet the various needs of preschool children during the parent–child relationship, and to some extent, it also reflects the perception of family cohesion. Relevant research findings have indicated that a favourable parenting sense of competence is capable of not only mitigating the parenting stress of parents but also effectively modulating their parenting behaviours and augmenting their involvement in the process of parenting (Trahan, 2017; Fang et al., 2021; Albanese et al., 2019). In this benign interaction, preschool children are provided with a more suitable developmental environment, exhibit more prosocial behaviours, and actively engage in social interactions (Salo et al., 2022).

In addition, a chain mediation model was verified. The influence of family cohesion on preschool children’s prosocial behaviour is realized by enhancing parenting sense of competence through positive parent–child relationships. This result can be explained by Bronfenbrenner’s ecological systems theory (Moen et al., 1995; Bronfenbrenner & Evans, 2000), which posits that children’s development is the product of bidirectional interactions between the individual and the environment. The environment has an impact on the child’s development. Conversely, the child’s development also exerts an influence on the environment. Regarding children aged 3–6 years, although the parent–child relationship between them and their parents is restricted by the micro-system, namely the family environment, this situation also has an effect on the parents within the family, especially their perception of their own parenting ability. This perception will ultimately affect the development of their children’s social behaviour, embodying the dynamic interaction between the individual and the environment. Notably, the current study further revealed that the individual mediating effect of parenting sense of competence is the most prominent, underscoring the unique significance of parenting sense of competence in the social development of young children. According to the interaction model, the psychological development of young children is affected by the continuous and dynamic interactions between young children and their caregivers. Specifically, parenting sense of competence has an impact on the social development of young children, which in turn influences their prosocial behaviour. On the other hand, the prosocial behaviour of young children also affects parenting sense of competence (Slagt et al., 2012).

### 4.3. Differences in Parental Roles

The innovative finding of this study is that the effect of family cohesion on the prosocial behaviour of preschool children varies by parents. The mediating effect of the key variable, parenting sense of competence, is significant only among fathers. This finding aligns with the actual situation of family education in China. In China, the key caregivers of young children are almost invariably mothers, whereas the role of fathers in parenting is considerably weak to the extent that single parenting, characterized by mothers serving as the almost exclusive primary caregivers due to weak involvement from fathers, has become a common occurrence. Previous research has indicated that, in China’s current family education, due to work stress, marital satisfaction, personal characteristics, role identification, or mothers’ gatekeeping behaviour, fathers’ willingness and level of participation in parenting are lower than those of mothers (Liu et al., 2022; Xu et al., 2019). Moreover, it has been confirmed that parenting efficacy is closely related to parental involvement (Pelletier & Brent, 2002). Research indicates that in China, greater father’s involvement in parenting is beneficial for fostering a child’s well-rounded personality, intellectual development, social skills, and independence (Tong, 2008). Father involvement in parenting is significantly positively correlated with children’s self-esteem and prosocial risk-taking behaviour (Wang, 2025). Considering the relatively low degree of father involvement, fathers demonstrate a reduced level of parenting sense of competence and show reluctance toward engaging in parenting activities. This leads to a decline in parenting effectiveness and ultimately exerts a substantial and far-reaching influence on the social development of young children.

### 4.4. Research Significance and Limitations

The results of this study can guide the promotion of children’s social development through family education. First, this study advances existing theoretical frameworks by proposing and testing an integrated chain mediation model that simultaneously examines parent–child relationships and parenting sense of competence as sequential mediators in the relationship between family cohesion and children’s prosocial behaviour. This approach extends beyond prior research, which typically examined these mediating variables in isolation, by revealing the dynamic and interconnected nature of family processes. Specifically, our findings demonstrate that strong family cohesion strengthens parenting sense of competence by enhancing the level of parent–child relationships and ultimately increases the occurrence of prosocial behaviour in children. Second, this study has uncovered the crucial significance of parenting sense of competence in the development of young children’s social behaviour. This further revealed the differences in the factors influencing young children’s prosocial behaviour between groups of fathers and mothers, underscoring the importance of “the father’s presence” in family education. We appeal for more Chinese fathers to invest in the education and development of their children.

This study is subject to certain limitations. The use of a single-item measure may not fully capture the complexity of the parent–child relationship and may have introduced measurement error, potentially affecting the precision of our estimated effects. And relying solely on parent reports for all measures may introduce shared method variance, which could affect the interpretation of our findings. This study exclusively analyzed the causal relationship between family cohesion and the prosocial behaviour of preschool children in the parent–child relationship and parenting sense of competence. There is a dearth of support from both longitudinal tracking data and educational experimental data. Future research may utilize educational experiments as an auxiliary measure.

## 5. Conclusions

The conclusions drawn from this study are as follows: (1) family cohesion can directly and notably affect the prosocial behaviour of preschool children, and stronger family cohesion can effectively increase children’s prosocial behaviour; (2) parent–child relationships and parenting sense of competence serve as significant mediators in the relationship between family cohesion and preschool children’s prosocial behaviour, and strong family cohesion strengthens parenting sense of competence by enhancing the level of parent–child relationships and ultimately increases the occurrence of prosocial behaviour in children; (3) further PSM analysis showed that the effect of family cohesion on the prosocial behaviour of preschool children varies by parents, with parenting sense of competence exhibiting notable significance within the group of fathers, while the parent–child relationship demonstrates considerable importance among mothers.

## Figures and Tables

**Table 1:** Correlations between the core variables.

	1	2	3	4
1 Family cohesion	1			
2 Parenting sense of competence	0.50 **	1		
3 Parent–child relationship	0.23 **	0.24 **	1	
4 Prosocial behaviours	0.45 **	0.37 **	0.29 **	1

Note: * *p* < 0.05, ** *p* < 0.01, *** *p* < 0.001; the same applies below.

**Table 2:** Direct and indirect path between family cohesion and prosocial behaviour.

Path	β	95% Confidence Interval
Direct effect		
Family cohesion → prosocial behaviour	0.31	[0.22, 0.40]
Mediating effect		
Family cohesion → parent–child relationship→ prosocial behaviour	0.04	[0.02, 0.06]
Family cohesion → PSOC → prosocial behaviour	0.07	[0.03, 0.11]
Family cohesion → parent–child relationship → PSOC → prosocial behaviour	0.01	[0.00, 0.01]

## Data Availability

The data presented in this study are available on request from the corresponding author due to privacy or ethical restrictions.

## Abbreviation

The following abbreviation is used in this manuscript:

PSOC	Parenting Sense of Competence
